# Mapping and validation of the epistatic *D* and *P* genes controlling anthocyanin biosynthesis in the peel of eggplant (*Solanum melongena* L.) fruit

**DOI:** 10.1093/hr/uhac268

**Published:** 2022-12-02

**Authors:** Qian You, Huimin Li, Jun Wu, Tao Li, Yikui Wang, Guangwen Sun, Zhiliang Li, Baojuan Sun

**Affiliations:** Guangdong Key Laboratory for New Technology Research of Vegetables, Vegetable Research Institute, Guangdong Academy of Agricultural Sciences, Guangzhou, Guangdong 510640, China; Guangdong Key Laboratory for New Technology Research of Vegetables, Vegetable Research Institute, Guangdong Academy of Agricultural Sciences, Guangzhou, Guangdong 510640, China; College of Horticulture, South China Agricultural University, Guangzhou, Guangdong 510642, China; Ganzhou Research Institute of Vegetables and Flowers, Gannan Academy of Sciences, Ganzhou, Jiangxi 341400, China; Guangdong Key Laboratory for New Technology Research of Vegetables, Vegetable Research Institute, Guangdong Academy of Agricultural Sciences, Guangzhou, Guangdong 510640, China; College of Horticulture, South China Agricultural University, Guangzhou, Guangdong 510642, China; Guangdong Key Laboratory for New Technology Research of Vegetables, Vegetable Research Institute, Guangdong Academy of Agricultural Sciences, Guangzhou, Guangdong 510640, China; Institute of Vegetable, Guangxi Academy of Agricultural Sciences, Nanning, Guangxi 530007, China; College of Horticulture, South China Agricultural University, Guangzhou, Guangdong 510642, China; Guangdong Key Laboratory for New Technology Research of Vegetables, Vegetable Research Institute, Guangdong Academy of Agricultural Sciences, Guangzhou, Guangdong 510640, China; Guangdong Key Laboratory for New Technology Research of Vegetables, Vegetable Research Institute, Guangdong Academy of Agricultural Sciences, Guangzhou, Guangdong 510640, China

## Abstract

Fruit color is an important trait influencing the commercial value of eggplant fruits. Three dominant genes (*D*, *P* and *Y*) cooperatively control the anthocyanin coloration in eggplant fruits, but none has been mapped. In this study, two white-fruit accessions (19 141 and 19 147) and their F_2_ progeny, with 9:7 segregation ratio of anthocyanin pigmented versus non-pigmented fruits, were used for mapping the *D* and *P* genes. A high-density genetic map was constructed with 5270 SNPs spanning 1997.98 cM. Three QTLs were identified, including two genes on chromosome 8 and one on chromosome 10. Gene expression analyses suggested that the *SmANS* on chromosome 8 and *SmMYB1* on chromosome 10 were the putative candidate genes for *P* and *D*, respectively. We further identified (1) a SNP leading to a premature stop codon within the conserved PLN03176 domain of *SmANS* in 19 141, (2) a G base InDel in the promoter region leading to an additional *cis*-regulatory element and (3) a 6-bp InDel within the R2-MYB DNA binding domain of *SmMYB1*, in 19 147. Subsequently, these three variations were validated by PARMS technology as related to phenotypes in the F_2_ population. Moreover, silencing of *SmANS* or *SmMYB1* in the purple red fruits of F_1_ (E3316) led to inhibition of anthocyanin biosynthesis in the peels. Conversely, overexpression of *SmANS* or *SmMYB1* restored anthocyanin biosynthesis in the calli of 19 141 and 19 147 respectively. Our findings demonstrated the epistatic interactions underlying the white color of eggplant fruits, which can be potentially applied to breeding of eggplant fruit peel color.

## Introduction

Eggplant (*Solanum melongena* L., 2n = 24) is a popular vegetable crop cultivated worldwide. In 2019, the total production of eggplant in China was 35.56 million tons, accounting for 64.48% of the global production (55.15 million tons) (https://www.tridge.com). Fruit peel color (hereafter referred to as fruit color) is one of the most important quality traits in eggplant and hence is a main breeding objective. Eggplant fruits have many colors including purple black, purple red, green, white, pink and others. Two types of pigments determine the fruit color of eggplants namely, anthocyanins, which are the main pigments biosynthesis via the flavonoid pathway, and chlorophylls [1]. Previous studies have shown that anthocyanin biosynthesis in eggplant fruit is controlled by three genes, symbolized as *D*, *P* and *Y* [2]. Similarly, an early study showed that the inheritance of color in potato was controlled by three genetic loci, namely *D*, *R* and *P*. Among these loci, *D* is a special developer related to tissue specificity of anthocyanin distribution. The red color depends on the *R* locus while the purple depends on *P* [3].

As a major factor determining fruit color, anthocyanin biosynthesis via a secondary metabolic pathway has been studied extensively in model plants, such as snapdragon, petunia, maize and *Arabidopsis* [4, 5]. Various structural genes have been reported to be involved in anthocyanin biosynthesis, including *chalcone synthase* (*CHS*), *chalcone isomerase* (*CHI*), *flavanone 3-hydroxylase* (*F3H*), *flavonoid 3′-hydroxylase* (*F3’H*), *flavonoid 3′5’-hydroxylase* (*F3’5’H*), *dihydro flavonol-reductase* (*DFR*), and *anthocyanin synthase* (*ANS*) [4, 6]. The expression of these genes is regulated by transcription factors such as MYB. Additionally, basic helix–loop–helix (bHLH) and WD repeat proteins (WDR) may form a MYB-bHLH-WDR (MBW) complex to regulate anthocyanin biosynthesis [7]. The solanaceous model plant, petunia has the largest number of studied MYB transcription factors related to regulation of anthocyanin biosynthesis, including *PhAN2*, *PhAN4*, *MYB27*, *DPL*, *PHZ*, and *PH4* [8–11]. Moreover, it was reported that over-expression of *SmMYB1* promoted anthocyanin biosynthesis in the peel and flesh of eggplant fruit [12].

It is well-documented that anthocyanin biosynthesis in the peel of eggplant fruit is controlled by multiple genes. Over the past decades, several studies have been reported on identification of quantitative trait loci (QTLs) associated with fruit color in eggplant. Nunome [13] constructed a linkage map using 88 RAPD (randomly amplified polymorphic DNA) and 93 AFLP (amplified fragment length polymorphism) markers and identified two QTLs associated with fruit color (anthocyanin biosynthesis) on the linkage group (LG) 7. Doganlar et al. mapped two QTLs associated with fruit color on the linkage groups 8 and 10 [14]. Subsequently, a pair of co-dominant AFLP and SCAR (sequence characterized amplified region) markers were suggested to be linked with the two fruit colors, dark purple and purplish red, respectively [15]. In a comprehensive study, a total of 415 SNP (single nucleotide polymorphism) markers were used to construct a linkage map of eggplant using an F_2_ mapping population bred from the cross “305E40” × “67/3”. The study revealed that eight QTLs associated with the trait of anthocyanin content of seven different plant parts (excluding fruits) were located on chromosomes 1, 5, 7, 10, and 11 [16]. Furthermore, a genome-wide association study (GWAS) showed that a series of SNP markers associated with fruit color were located on chromosomes 1, 5, 7, 10, and 11 [17]. Recently, several QTLs and QTNs (quantitative trait nucleotides) controlling fruit anthocyanin pigmentation were reported to be co-localized with the selective sweeps on chromosome 10, and a total of 15 candidate genes were identified within the selective sweep regions for the fruit pigmentation [18].

In the present study, crossing two accessions with white fruits led to an F_1_ (E3316) population with purple red fruits. Moreover, the segregation ratio of plants with anthocyanin-pigmented fruits to those with non-anthocyanin-pigmented fruits was 9:7 in the F_2_ population. This result indicated that the inheritance of fruit color in these two parental lines was controlled by two pairs of completely dominant genes with epistatic interaction [19]. So far, the mechanism of how epistatic genes in the anthocyanin biosynthesis pathway affect the pigment contents of eggplant fruits is still unclear.

The current study aimed to (1) gain better understanding of the epistatic inheritance of fruit coloration, (2) provide novel molecular markers for breeding varieties with different fruit colors, and (3) facilitate elucidating the mechanism of epistatic gene interaction in regulating the fruit color in eggplant. Thus, QTL mapping was used to identify the epistatic QTL regions controlling anthocyanin biosynthesis in the F_2_ population. Mutations in the candidate genes were identified, which could be responsible for the lack of anthocyanin in the fruits of the two parental lines, 19 141 and 19 147. Subsequent functional analysis and characterization of these two candidate genes confirmed their role in anthocyanin biosynthesis in eggplant fruits.

## Results

### Inheritance of fruit color in the F_2_ population is controlled by the two epistatic loci *D* and *P*

**Figure 1:**
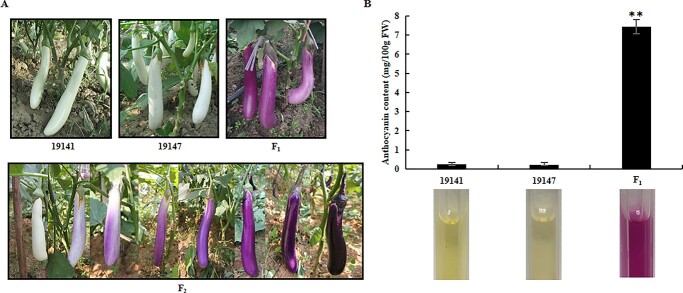
Fruit color (A) and anthocyanin contents (B) of the eggplant parental lines 19 141 and 19 147, the F_1_ (E3316) and the F_2_ progeny. (^**^, *p* < 0.01).

If two dominant genes control anthocyanin biosynthesis in the peels of eggplant fruits, a homozygous recessive mutation at either of the gene loci would inhibit anthocyanin biosynthesis. The fruits of the two inbred lines 19 141 and 19 147 were white, while those of 30 (100.00%) F_1_ (E3316) hybrids were purple red (Fig. 1A). Subsequently, two F_2_ populations, comprising 1591 (population 1) and 486 (population 2) F_2_ progeny showed segregation of the fruit color. Then, we measured the total anthocyanin contents of the fruit peels in 19 141 and 19 147 as well as F_1_ (E3316). The results showed that the anthocyanin content was 7.43 mg/100 g FW in the F_1_ plants, whereas it was minimal in 19 141 (0.24 mg/100 g FW) and 19 147 (0.21 mg/100 g FW) (Fig. 1B).

The chi-square test was used to check the fitness of the segregation ratio of the anthocyanin pigmented and white fruit genotypes in the F_2_ populations, where it showed 9:7 ratio with *p* > 0.05 (0.19 and 0.73, respectively) (Table 1). The segregation results implied that there were possibly two natural recessive mutations in the anthocyanin biosynthesis genes *P* and *D* in the lines 19 141 and 19 147, respectively. Such mutations may have resulted in the white fruit phenotypes (non-anthocyanin), for which the alleles could complement one another to produce the anthocyanin-pigmented fruits in the F_1_ plants. Additionally, since anthocyanin were accumulated in different parts of these two parental plants, we speculated that the genotype of 19 141 was *DDp^w^p^w^* with no anthocyanin pigmentation in all plant parts, which could be caused by mutation of the structural gene*.* Consequently, the genotype of 19 147 was *d^t^d^t^PP* with non-anthocyanin pigmented fruits and tinged anthocyanin in the flower, which could be caused by mutation of the regulatory gene (Fig. S1). Moreover, theoretically, all F_1_ (E3316) progeny was entirely *Dd^t^Pp^w^* and produced four types of gametes forming nine genotypes in the F_2_ population (Table S1), among which five genotypes (*DDp^w^p^w^*, *Dd^t^p^w^p^w^*, *d^t^d^t^PP*, *d^t^d^t^Pp^w^,* and *d^t^d^t^p^w^p^w^*) constituted the white fruit progeny while the remaining four (*DDPP, DDPp^w^, Dd^t^PP,* and *Dd^t^PP*) represented the anthocyanin-pigmented fruits.

**Table 1:** Segregation ratios of anthocyanin pigmented and non-pigmented fruits in the parents, F_1_ (E3316) and F_2_ populations

**Plant type**	**Total No.**	**Anthocyanin pigmented**	**non-pigmented**	**χ** ^**2**^ **(9:7)**	***P* value**
19 141 female	30	-	30	-	-
19 147 male	30	-	30	-	-
F_1_ progeny (E3316)	30	30	0	-	-
F_2_ population 1	1591	888	703	0.12	0.73
F_2_ population 2	486	259	227	1.72	0.19

### Identification of the epistatic QTLs and candidate genes for *D* and *P*

Since the size of F_2_ population 1 (1591) was too large to maintain, we scored the phenotypes of fruit colors of this population only, and planted the F_2_ population 2 (486) for genotyping in the next season. A total of 200 plants from F_2_ population 2 were randomly selected for genotyping, including 88 (44.00%) with white fruits and 112 (56.00%) with anthocyanin-pigmented fruits (Table S2). In total, 477 640 specific length amplified fragment (SLAF) tags were obtained, which generated 141.08 Gb of data comprising 683.46 Mb paired-end reads. Out of these reads, 93.60% passed Q30, and the guanine cytosine (GC) content was 39.47%. Then, GATK and Samtools softwares were both used to finally identify 2 712 851 common SNPs, of which 2 276 556 (83.92%) were polymorphic between the two parental genotypes. Among these polymorphic SNPs, 417 117 (15.38%) SNPs with aa×bb pattern were used for further analysis. After quality screening and filtering, a total of 6295 SNP markers were obtained, which were used to construct linkage maps with HighMap [20]. The average sequencing depth of these SLAF markers on the genetic map was 15.73-fold in the F_2_ individuals. Finally, out of the 6295 SNP markers, 5270 (83.72%) SNPs (Table S3) were successfully mapped to 12 LGs, covering 1997.98 cM genetic distance with an average marker density of 0.38 cM/marker (Table 2, Fig. S2). The number of SNP markers on each LG ranged from 121 (LG2) to 1795 (LG7). Similarly, the genetic distance of each LG spanned from 136.88 cM (LG6) to 193.48 cM (LG1), and the average marker intervals ranged from 0.10 cM (LG7) to 1.28 cM (LG9). Additionally, the longest linkage group was LG1, where it contained 161 SNPs, whereas the shortest one was LG6, comprising 807 SNPs (Table 2).

**Table 2:** Summary of the linkage map for the F_2_ population of the cross 19 141 × 19 147

Linkage group	Total marker no.	Total distance (cM)	Average distance (cM)	Maximum gap (cM)	Gap <5 cM
1	161	193.48	1.20	7.30	97.50%
2	121	137.80	1.14	9.16	98.33%
3	551	180.35	0.33	11.37	98.91%
4	162	178.46	1.10	12.46	98.14%
5	610	166.27	0.27	6.20	99.51%
6	807	136.88	0.17	6.60	99.75%
7	1795	188.30	0.10	3.41	100.00%
8	250	154.69	0.62	15.20	99.20%
9	122	156.74	1.28	9.11	92.56%
10	366	186.68	0.51	4.71	100.00%
11	189	167.89	0.89	8.39	98.40%
12	136	150.44	1.11	11.59	97.78%
Total	5270	1997.98	0.38	15.20	98.34%

**Figure 2:**
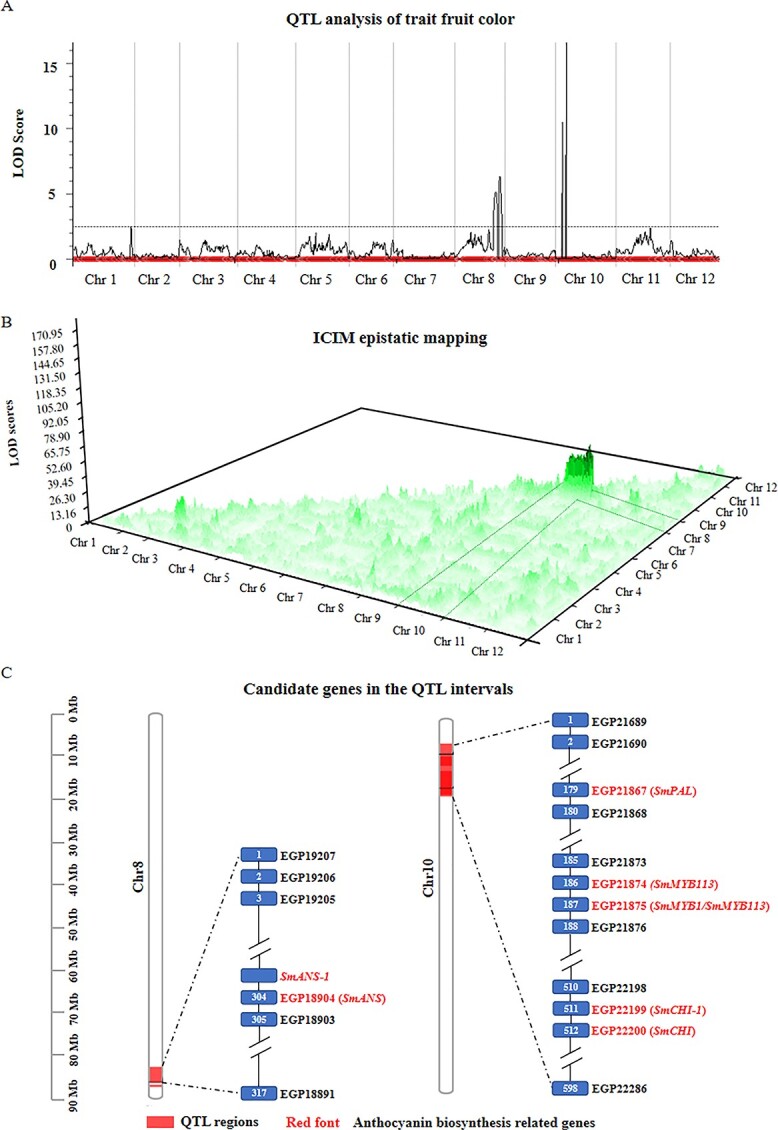
QTL mapping of the fruit color of eggplant and identification of candidate genes related to anthocyanin biosynthesis in QTL intervals. (A) The QTL peaks and their corresponding covered areas related to fruit color on chromosomes 8 and 10; (B) Detection of epistatic QTL intervals using ICIM-EPI (epistasis) mapping method; (C) Identification of candidate genes related to anthocyanin biosynthesis in the corresponding QTL intervals based on GUIQIE-1 genome.

The ICIM method was used to identify four QTLs associated with fruit color in the F_2_ population (Fig. 2A). The two QTLs qfcol8.126 and qfcol8.139 were located to chromosome 8 with PVE values of 7.57% and 8.54% and LOD values of 5.12 and 6.31, respectively (Table 3). The other two QTLs qfcol10.21 and qfcol10.33 were mapped to chromosome 10, which explained 25.20% (LOD = 17.12) and 14.26% (LOD = 10.48) phenotypic variance, respectively (Table 3). Then the ICIM-EPI (epistasis) mapping method was used to detect the epistatic QTL intervals. From a three-dimensional ICIM-EPI mapping at all scanning positions, the QTL intervals on chromosome 8 strongly interacted with the QTL intervals on chromosome 10 (Fig. 2B).

**Table 3:** Quantitative trait loci (QTL) associated with fruit color in the F_2_ population based on IciMapping software results

**QTL ID**	**Chr.**	**Pos.**	**Left marker**	**Right marker**	**Start (cM)**	**End (cM)**	**LOD**	**PVE**	**ADD**	**DOM**
qfcol8.126	8	126	Marker422646	Marker855400	136.41	140.94	6.31	8.54	−0.19	0.01
qfcol8.139	8	139	Marker335655	Marker740984	119.95	128.93	5.12	7.57	−0.06	0.25
qfcol10.21	10	21	Marker693671	Marker806826	20.74	21.49	10.48	14.26	0.02	0.34
qfcol10.33	10	33	Marker1149956	Marker1127935	31.46	33.12	17.12	25.20	0.32	0.01

Note: Chr.: chromosome; Pos.: marker position on map; LOD: logarithm of odds; PVE: phenotypic variance explained; ADD: additive effect; DOM: dominance effect.

The above QTL data suggested that the candidate genes related to fruit color are located on chromosomes 8 and 10. To search for genes within these identified QTL regions on the two chromosomes, the four QTL regions (qfcol8.126, qfcol8.139, qfcol10.21 and qfcol10.33) were further analyzed. Due to the availability of a high-quality reference genome at the time of searching for candidate genes, the sequences of the markers flanking these QTL regions were mapped to the chromosome-scale genome of eggplant GUIQIE-1 [21] to identify the corresponding QTL regions (Table S4). The annotated genes within these genomic regions were screened to identify the candidate genes. As a result, a total of 317 and 34 genes were found within the two QTL intervals qfcol8.126 and qfcol8.139 on chromosome 8, among which two genes were related to anthocyanin biosynthesis in qfcol8.126 (Fig. 2C, Table S5). Moreover, a total of 598 and 50 genes were identified within the intervals of qfcol10.21 and qfcol10.33 on chromosome 10, where the interval of qfcol10.33 was included in the qfcol10.21 according to the GUIQIE-1 reference and hence they could be considered as single QTL interval (qfcol10.21). Then, a total of five genes associated with anthocyanin biosynthesis was annotated within the qfcol10.21 (Fig. 2C, Table S5).

### Identification of two candidate genes controlling anthocyanin biosynthesis

To explore whether the above seven genes associated with anthocyanin biosynthesis have expression differences or sequence mutations, we analyzed our previous RNA-seq data of RNA samples extracted from fruit peels of the two parental lines and F_1_ (E3316) progeny with three biological replicates, at the marketable fruit stage [22]. Analysis of the FPKM values of these seven genes in the QTL regions revealed that the two genes EGP21874 (*SmMYB113*) and EGP22199 (*SmCHI-1*) showed no expression in 19 141 or 19 147, while two other genes, Chr08:87458149–87 459 791 (*SmANS-1*) and EGP21867 (*SmPAL*), exhibited no significant differences among the two parents and the F_1_ (E3316). Contrarily, the remaining three genes EGP18904 (*SmANS*), EGP21875 (*SmMYB1*) and EGP22200 (*SmCHI*) displayed significant differences among the two parents and the F_1_ (E3316) (Table S6)*.* More specifically, EGP18904 (*SmANS*), EGP21875 (*SmMYB1*) and EGP22200 (*SmCHI*) had significantly lower expression levels in the line 19 147 compared to 19 141 (0.29 vs 238.1, 0 vs 16.27, and 0.33 vs 43.58), respectively. To check for sequence polymorphisms in the promoters or coding regions of the three differentially expressed genes (DEGs) in the two parental plants, the in-house available whole genome re-sequencing data of 19 141 and 19 147 were analyzed. The results showed no sequence polymorphisms in the promoter regions of EGP22200 (*SmCHI*), EGP18904 (*SmANS*), except a G/A SNP and a G base InDel in EGP21875 (*SmMYB1*) between the two parental lines. No sequence polymorphisms were detected in the coding region of EGP22200 (*SmCHI*). Remarkably, a SNP leading to a premature stop codon at a conserved domain was identified in EGP18904 (*SmANS*) of 19 141 and an Indel (6 bp-codon deletion) at the R2-MYB DNA binding domain was identified in EGP21875 (*SmMYB1*) of 19 147. In view of the above data, we suggested that EGP18904 (*SmANS*) and EGP21875 (*SmMYB1*) may be the target candidate genes.

### qRT-PCR validation of *SmANS* and *SmMYB1*

**Figure 3:**
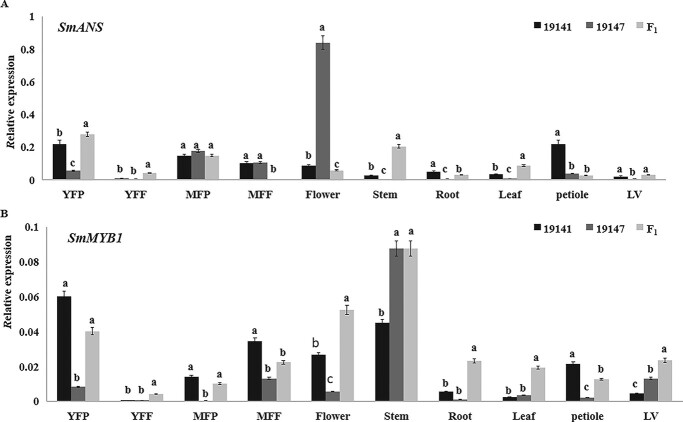
The expression levels of the candidate DEGs in different tissues from the two parental lines and F_1_ (E3316). (A) *SmANS* expression. (B) *SmMYB1* expression. YFP: young fruit peel; YFF: young fruit flesh; MFP: marketable fruit peel; MFF: marketable fruit flesh; LV: leaf vein. Different lowercase letters indicate statistical differences as shown by multiple range test (*p* ≤ 0.05) of variance (ANOVA) method.

The tissue-specific expression of the two selected candidate DEGs *SmANS* and *SmMYB1* was analyzed by qRT-PCR. *SmANS* was expressed to a significantly higher level in the fruit peel of 19 141 than in that of 19 147 (Fig. 3A). In addition, *SmANS* was mainly expressed in the flowers of 19 147 (Fig. 3A). The expression levels of *SmMYB1* in the young and marketable fruit peels of the female parent (19141) were significantly higher than those in the male one (19147) in which the expression levels were very low or undetectable (Fig. 3B). The expression level of *SmMYB1* in the F_1_ (E3316) was intermediate with respect to the two parental lines, indicating that the expression of this gene was altered in 19 147. In addition, *SmMYB1* was highly expressed in the flesh of the marketable fruits and flowers of 19 141 and to even higher levels in the stem, vein and flesh of the marketable fruits of 19 147 (Fig. 3B). The trend of *SmMYB1* expression levels in the F_1_ (E3316) was similar to that of 19 147, suggesting that the expression level of *SmMYB1* was positively correlated with accumulation of anthocyanins in eggplant fruits. *SmMYB1* also showed varying expression patterns in different tissues at different developmental stages.

### Isolation and analysis of *SmANS* and *SmMYB1*

**Figure 4:**
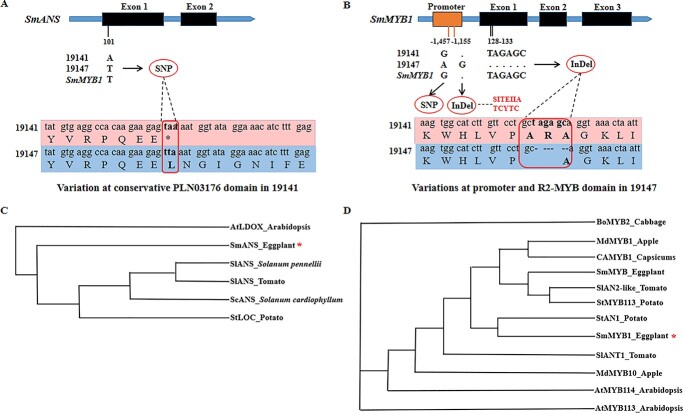
Variation in the conserved domains and phylogenetic analysis of *SmANS* and *SmMYB1*. (A) A SNP on exon1 led to a stop codon at the PLN03176 domain of *SmANS* in 19 141; (B) A six-nucleotide deletion on exon1 caused shrinking of *SmMYB1* by two amino acids at R2 MYB domain in 19 147; (C) and (D) Phylogenetic analysis for *SmANS* and *SmMYB1* from *Solanum melongena* L. and the orthologs from other species. ^*^ represents the studied genes in the present work.

To validate the mutations of the two candidate genes, the amplicon sequences of *SmANS* and *SmMYB1* were compared in 19 141 and 19 147, respectively. *SmANS* contained a full-length gDNA of 3129 bp including an open reading frame (ORF) of 1242 bp with two exons and one intron (Fig. 4A). The data revealed that the nucleotide number 101 of *SmANS* coding region was mutated from T to A in 19 141, leading to TAA, a premature stop codon at nucleotides number 101 and 102 in the conserved PLN03176 domain. The peptide length of *SmANS* in 19 141 was predicted to be truncated from 413 to 33 amino acids (Fig. 4A). Thus, we speculated the loss of *SmANS* (*P* gene) function resulted in the white fruit phenotype (non-anthocyanin) in 19 141.

For *SmMYB1*, the data revealed that it had a full-length gDNA of 1230 bp with an ORF of 771 bp comprising three exons and two introns (Fig. 4B). A SNP (19141G: 19147A) at −1457 bp upstream and a G base InDel (19 141/: 19147G) at −1155 bp upstream of the start codon were identified, respectively. According to the PLACE analysis (https://www.dna.affrc.go.jp/PLACE/?action=newplace), the G base InDel (−1155 bp) was predicted to lead to an additional *cis*-regulatory element SITEIIATCYTC (TGGGCY) in 19 147, which may have down-regulated *SmMYB1* in this line. Moreover, a 6-bp nucleotide deletion was detected at 128–133 bp in the coding region of the first exon of *SmMYB1* in 19 147, resulting in the lack of two amino acids. Thus, the length of the peptide was predicted to be shrunk from 256 amino acids to 254 amino acids in 19 147 (Fig. 4B). Importantly, these two amino acids were located at the R2-MYB DNA binding domain, which was highly conserved in transcription factors involved in the control of anthocyanin biosynthesis. Therefore, we speculated that the additional *cis*-regulatory element in the promoter region and the disruption of the DNA binding domain could have potentially diminished the expression or influenced the function of *SmMYB1* (*D* gene), resulting in white fruits in 19 147.

Based on the amino acid sequences, a phylogenetic tree containing orthologs of *SmANS* from several species (including some solanaceous ones), was constructed (Fig. 4C). The results showed that compared with other genes, the *SmANS* in eggplant was closer to *StLOC* in Potato, but was distant from *AtLDOX* in *Arabidopsis.* The orthologs of *SmMYB1* from the above species were used to construct a phylogenetic tree according to their amino acid sequences (Fig. 4D). As expected, data on *SmMYB1* cloned from 19 141 were consistent with previous reports, where it clustered with the *StAN1* from potato.

### Development of PARMS markers and validation of the *SmANS* and *SmMYB1* in the F_2_ population

**Figure 5:**
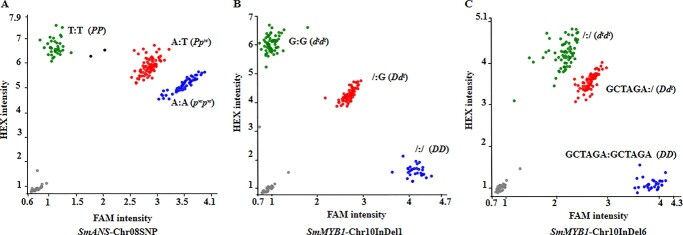
PARMS genotyping variations of *SmANS* and *SmMYB1* in the F_2_ population. (A) PARMS genotyping results for primers of *SmANS*-Chr08SNP. FAM intensity (A:A) corresponds to *p^w^p^w^*, FAMHEX intensity (A:T) corresponds to *PP*, and HEX intensity (T:T) corresponds to *Pp^w^*. (B) and (C) PARMS genotyping results for primers of *SmMYB1*-Chr10InDel1 (B) and *SmMYB1*-Chr10InDel6 (C). FAM intensity corresponds to *DD*, FAMHEX intensity corresponds to *Dd^t^*, and HEX intensity corresponds to *d^t^d^t^*.

To validate the identified variations of *SmANS* and *SmMYB1* in the F_2_ population, and to test the genotype–phenotype associations, a different set of 200 F_2_ progeny were genotyped at the three loci, *SmANS*-Chr08SNP (Fig. 5A), *SmMYB1*-Chr10InDel1 (Fig. 5B), and *SmMYB1*-Chr10InDel6 (Fig. 5C) through PARMS (Penta prime amplification reflex mutation system) technology [23]. The data showed that the genotyping results of the two loci of *SmMYB1*, *SmMYB1*-Chr10InDel1 and *SmMYB1*-Chr10InDel6, were identical (Table S7). Out of 50 F_2_ progeny with white flowers and white peels, which were all *p^w^p^w^* (*Smans*), 24 F_2_ plants had the *Dd^t^p^w^p^w^* genotype, 17 had the *d^t^d^t^p^w^p^w^* genotype and the remaining 9 had *DDp^w^p^w^*. Among the other 50 F_2_ progeny with purple flowers and white peels, which were all *d^t^d^t^* (*Smmyb1*), 16 plants were *d^t^d^t^PP* and 34 were *d^t^d^t^Pp^w^*. Out of 100 F_2_ progeny with purple flowers and purple red peels, 7 were *DDPP*, 22 were *DDPp^w^*, 18 were *Dd^t^PP*, and 52 were *Dd^t^Pp^w^*. One plant was NTC (negative control).

The above results demonstrate that four genotypes (*DDPP*, *DDPp^w^*, *Dd^t^PP* and *Dd^t^Pp*) corresponded to the purple red peels phenotype and five genotypes (*DDp^w^p^w^*, *Dd^t^p^w^p^w^*, *d^t^d^t^PP*, *d^t^d^t^Pp^w^* and *d^t^d^t^p^w^p^w^*) corresponded to the white peels. This indicates that the genetic variations in *SmANS* and *SmMYB1* were completely in line with phenotypes of peel color in the F_2_ population. In addition, the results suggested that the structural gene *SmANS* was related to anthocyanin biosynthesis in all parts of the plant, while the regulatory gene *SmMYB1* was related to tissue specificity of anthocyanin biosynthesis.

### VIGS and overexpression of *SmANS* and *SmMYB1*

**Figure 6:**
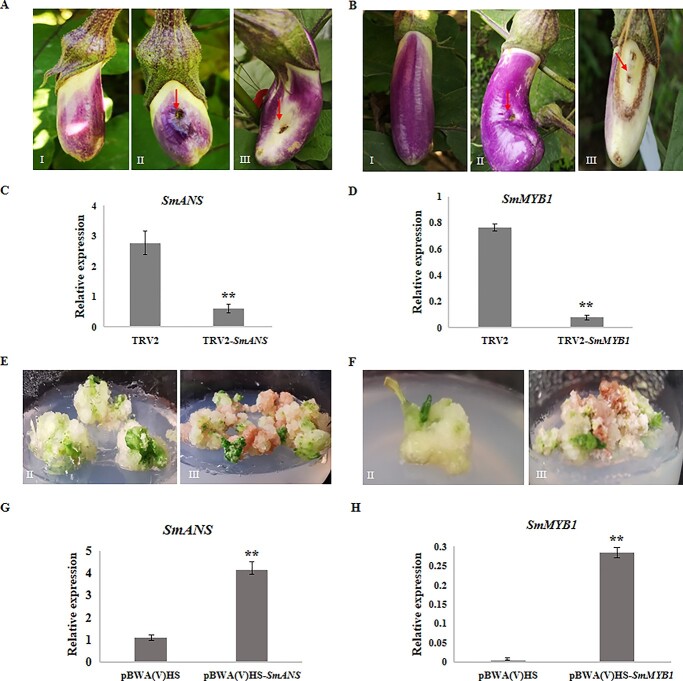
The phenotypes and expression levels in eggplant fruits infiltrated with different VIGS constructs and overexpression of *SmANS* and *SmMYB1* in eggplant calli. (A) and (B) Phenotypes of the F_1_ (E3316) fruits infiltrated with different VIGS constructs for *SmANS* and *SmMYB1*, including (I) normal fruits, (II) TRV-empty vector, and (III) TRV-*SmANS* (A) and TRV-*SmMYB1* (B). Red arrows show the infiltrated positions. (C) and (D) the expression levels of *SmANS* and *SmMYB1* in VIGS-fruits of F_1_ (E3316). (E) and (F) Non-anthocyanin calli transformed with pBWA(V)HS-empty vector (E,II and F, II), and pigmented calli transformed with pBWA(V)HS-*SmANS* (E, III) in 19 141 and pBWA(V)HS-*SmMYB1* (F, III) in 19 147. (G) The expression levels of pBWA(V)HS-*SmANS* in transgenic calli of 19 141; (H) The expression levels of pBWA(V)HS-*SmMYB1* in the transgenic calli of 19 147.

To validate the function of *SmANS* in anthocyanin biosynthesis in eggplant, we infiltrated the 1 cm young fruits of the F_1_ (E3316) (4 to 6 days after pollination) with GV3101 *Agrobacterium* cultures harboring pTRV2-*SmANS* mixed with *Agrobacterium* carrying pTRV1 in the ratio 1:1, using a syringe. One week after inoculation, the fruits showed photo-bleaching around the infiltrated area due to *SmANS* silencing (Fig. 6A). Compared with the expression of *SmANS* gene (2.77) in eggplant fruits with pTRV2-empty vector (anthocyanin pigment), the fruits inoculated with pTRV2-*SmANS* showed down-regulated expression of *SmANS* (reduced to 0.61) as revealed by qRT-PCR analysis (Fig. 6C). Furthermore, to study the function of the *SmANS* gene, the full-length coding sequence of *SmANS* driven by the CaMV 35S promoter was overexpressed in the calli of 19 141. Compared with the hygromycin resistant calli transformed with the empty vector, the transgenic calli containing the *SmANS* gene vector-pBWA(V)HS-*SmANS* showed the phenotype of anthocyanin accumulation (Fig. 6E) and a significantly higher expression than that in vector-pBWA(V)HS (empty vector) (Fig. 6G). Therefore, both VIGS and overexpression experiments confirmed that the loss of *SmANS* function could be one of the reasons for the lack of anthocyanin biosynthesis in the fruit peels of 19 141. This indicated that *SmANS* is most likely the *P* gene.

We also validated the function of *SmMYB1* in eggplant by using GV3101 *Agrobacterium* cultures carrying pTRV2-*SmMYB1* mixed with bacteria carrying pTRV1 in a ratio of 1:1 to inoculate the young fruits of F_1_ plants (E3316) (4 to 6 days after pollination). After 7 days, the fruits showed photo-bleaching around the infiltrated area due to *SmMYB1* silencing (Fig. 6B). Then, the qRT-PCR of *SmMYB1* transcripts in the fruits with pTRV2-*SmMYB1* showed that the expression was much lower (0.08 vs 0.76) than that in the fruits inoculated with pTRV2-empty vector (Fig. 6D). Subsequently, the function of the *SmMYB1* gene was further studied where the *SmMYB1* coding sequence driven by the CaMV 35S promoter was overexpressed in the calli of 19 147. The results showed that, compared with calli inoculated with the empty vector, the transgenic calli inoculated with the vector-pBWA(V)HS-*SmMYB1* containing the *SmMYB1* gene, showed significant anthocyanin accumulation (Fig. 6F) as well as a significantly higher expression level than that of the control (empty vector) (Fig. 6H). Therefore, combined with the genotype–phenotype association results of PARMS markers, the VIGS and overexpression experiments verified that the disruption of *SmMYB1* could be one of the reasons for the lack of anthocyanin accumulation in the fruit peels of 19 147, suggesting *SmMYB1* as a candidate of the *D* gene.

## Discussion

Anthocyanins, a type of flavonoids, exist in eggplant fruits conferring very attractive colors. However, little is known about the molecular control of anthocyanin biosynthesis in eggplant fruits or how genes interact to regulate such phenotype. In this study, we adopted a forward genetics-QTL mapping approach to identify epistatic QTLs associated with anthocyanin biosynthesis in eggplant fruits and to validate the functions of two putative candidate genes by VIGS and overexpression assays. The present study provides a better understanding for the mechanisms of gene interaction in anthocyanin biosynthesis that affect the peel color of eggplant fruits.

### High-resolution genetic linkage map for epistatic gene mapping

Specific length amplified fragment sequencing (SLAF-seq) is a cost-effective sequencing strategy based on the improvement of reduced representation libraries (RRLs), which has been proven to be a high-throughput genotyping and variation discovery [24]. Based on SLAF-seq, we developed high-resolution (5270 mapped SNPs, 0.38 cM/SNP) genetic linkage maps of eggplant, as compared to the previously reported ones, where the number of markers ranged from 250 to 2122, and the marker density was 5.66 to 0.72 cM/marker [16, 25–32]. In addition, the linkage map constructed in our study was more saturated than that (2122 mapped SNP, 0.72 cM/SNP) constructed via the same sequencing method (SLAF-seq) [32]. The reason for this difference may be due to different population types and sizes. More specifically, an intraspecific F_2_ population comprising 200 plants was used in our study, whereas an interspecific F_2_ population containing 121 progeny was analyzed in the study of Wei et al. [32].

### Mapping of the epistatic genes *D* and *P*

QTL mapping is a useful approach to understand the genetic architecture of quantitative traits. Epistasis was first discovered in pea hybridization experiments. When two white-flowered parents were crossed, the flower color in the F_1_ was purple, and the segregation ratio of purple-flowered plants to the white-flowered ones was 9:7 in the F_2_ population. In order to explain this phenomenon, Bateson proposed the concept of epistasis which represented a deviation from the Mendelian genetic law [33]. Later, using the genome of a model legume as a reference, the genes *A* and *A2* were found to be related to anthocyanin pigmentation in pea [34]. The *A* gene encodes a bHLH transcription factor, and a simple G to A transition in a splice donor site in the white flowered-mutant led to a mis-spliced mRNA with a premature stop codon. The *A2* gene encodes a WD40 protein, which has a variety of mutant forms, resulting in white flowers.

In our study, it was considered that the interaction of two epistatic genes (*D* and *P*) controlled the color of eggplant fruit. We identified two QTLs (qfcol8.126 and qfcol8.139) on chromosome 8 and one (qfcol10.21) on chromosome 10 associated with fruit color in the F_2_ mapping population. Subsequently, epistatic QTL regions were also identified on chromosomes 8 and 10, which indicated that there were strong epistatic interactions between chromosomes 8 and 10 (Fig. 2B). Doganlar et al. [14] reported two QTLs (fc8.1 and fc10.1) related to fruit color on chromosomes 8 and 10 in an interspecific F_2_ population resulting from crossing *S. linnaeanum* MM195 (green fruit) and *S. melongena* MM738 (purple fruit). The flanking sequences of the five markers of the fc8.1 and fc10.1 intervals were aligned to the GUIQIE-1 genome, where one significant marker TG510 was in the qfcol8.126 interval and was close to the candidate gene *SmANS* (1.50 Mb), while the other significant marker CT240 was close to the qfcol10.21 interval but far from the candidate gene *SmMYB1* (12.65 Mb).

Additionally, several QTLs and QTNs controlling fruit anthocyanin pigmentation co-localized with the selective sweeps on chromosome 10 [18]. However, other studies have identified QTLs associated with fruit color on other chromosomes, such as chromosomes 1, 5, 7, 10, 11, and 12 [13, 15–17]. Since anthocyanin biosynthesis is a complex pathway that involves multiple structural and regulatory genes, a single mutation in any of the involved genes could prevent anthocyanin accumulation. This could be the reason for identification of various QTLs/genes on different chromosomes related to eggplant fruit color. Interestingly, chromosome 10 was the common chromosome to which QTLs related to fruit color in different mapping populations were mapped, indicating that the QTLs on this chromosome are more likely to be the broad-spectrum QTLs for anthocyanin pigmentation in eggplant fruit peel.

### Screening of candidate genes for *D* and *P*

Anthocyanin biosynthesis in fruit peels of potato, another *solanaceous* plant, is controlled by the loci *D*, *P* and *R*. It was proven that the *P* locus in potato encodes *F3’5’H*, the *R* locus encodes *DFR* [35], while the *D* locus on chromosome 10 was identified as the *StAN2* gene encoding an *R2R3-MYB* transcription factor [36]. The potato developer (*D*) locus also encodes the R2R3 MYB transcription factor *StAN2* [36], which was cloned from the bark of the tuber of the red potato genotype Y83–5. *StAN2* was expressed in the purple or red pericarp in the context of a dominant *D/I* gene, but not in the white pericarp under the recessive *dd* control. In addition, *StAN2* has been proven to significantly activate *DFR* and *F3’5’H* promoters according to dual-luciferase assay [36]. Similarly, we propose that there are two epistatic genes responsible for fruit color in eggplant, which were located to chromosomes 8 and 10. Through searching the corresponding QTL regions based on the annotation information in eggplant reference genome GUIQIE-1, combined with gene expression information and sequence polymorphism analysis, we hypothesized that *SmANS* on chromosome 8 and *SmMYB1* on chromosome 10 were candidate genes for *P* and *D*, respectively.

Comparing cDNA and predicted amino acid sequences of the two parental lines, a premature stop codon mutation in an exon of *SmANS* resulted in truncating the peptide from 413 to 33 amino acids. This may have inhibited anthocyanin biosynthesis in 19 141 fruits with the genotype of *DDp^w^p^w^.* Recently, through fine mapping of an F_2_ eggplant population, *SmFAS* (*SmANS*) was identified as a putative candidate gene containing four SNPs and one InDel in the coding region, of which the one nucleotide deletion resulted in premature termination of *ANS*, leading to the loss of anthocyanin accumulation in the flower [37].

In the current study, a G base InDel in the promoter at −1155 bp upstream may have led to the down-regulation of *SmMYB1* in 19 147. Additionally, a six-nucleotide deletion in the first exon of *SmMYB1* in 19 147 have resulted in loss of the amino acids number 43 and 44 within the R2-MYB domain, which could possibly disrupt the transcription activation function of the MYB protein [38] and eventually undermine or even block the anthocyanin biosynthesis in the fruits with the genotype of *P_d^t^d^t^*. It was reported that SNP polymorphisms in the promoter of *Awn Length Inhibitor 1* (*ALI-1*) led to the absence of the *cis*-elements BOXCPSAS1, LTRE1HVBLT49, SORLIP2AT and SITEIIATCYTC in wheat [39], resulting in up-regulation of *ALI-1* in the developing spike of awnless individuals and the decrease in grain length. Moreover, a 6 bp-deletion at the end of exon 1 of the *SmMYB1* gene was detected in eggplant accessions with green fruits (non-anthocyanin pigmentation) as DNA markers of disorders in anthocyanin biosynthesis [40]. This is consistent with our mutation data in *SmMYB1* coding region. Therefore, combined with the genotype–phenotype association results of PARMS markers, we speculated that the variations in the promoter of the white-peel 19 147 may play a role in the down-regulation of *SmMYB1.* Furthermore, the 6 bp-deletion in the R2-MYB domain may have impaired its ability to bind the target structural genes. However, such hypothesis requires further future experimental verification. Additionally, the developed PARMS markers were used to examine the consistency between genotypes and phenotypes in the F_2_ population, which also confirmed that variations of *SmANS* and *SmMYB1* genes could be the genetic reasons for the white fruit peel phenotype of the F_2_ population.

### Function of *SmANS* and *SmMYB1* in anthocyanin biosynthesis in eggplant fruit peel

Up to date, some enzyme-encoding genes such as *SmCHS*, *SmCHI*, *SmF3H*, *SmANS*, and *SmDFR* [41], as well as regulatory genes, such as *SmMYB1* [12], *SmMYB86* [42], *SmbHLH13* [43], involved in anthocyanin biosynthesis in eggplant fruits have been cloned and validated independently. A single base pair deletion (InDel) at the site 438 was reported to resulted in premature termination of *FAS* (*SmANS*), leading to loss of anthocyanin biosynthesis in white flowered-eggplant [37]. In our study, *SmANS* as a candidate gene for the *P* locus controlling fruit color also controls anthocyanin biosynthesis in other parts of the plant including the flower. Due to the nonsense mutation of *SmANS*, there was no anthocyanin accumulation in any part of the 19 141 plants.

The regulatory genes *SmMYB1/SmMYB113* have been cloned and demonstrated to play an important role in anthocyanin biosynthesis in eggplant fruits [12, 42]. However, identification of the naturally occurring polymorphism in *SmMYB1* associated with fruit color in eggplant through QTL mapping has not been reported. It has been reported that overexpression of *SmMYB1* in eggplant [12] promoted anthocyanin accumulation in calli, peels and pulps, which is consistent with our results. It is noteworthy that the anthocyanin color of the calli in our study was lighter than that of *SmMYB1* overexpressing calli in a previous report [12], presumably due to the different types of anthocyanins involved [44].

## Conclusion

Using special and representative parental lines and an F_2_ population, we mapped the epistatic genes *P* and *D* to chromosomes 8 and 10, respectively, based on a forward genetics approach. RNA-seq and whole genome re-sequencing data were used to narrow down the list of candidate genes, where the structural gene *SmANS* and the transcription factor gene *SmMYB1* were identified. Subsequently, PARMS markers were developed to correlate fruit color phenotypes with their corresponding genotypes in the F_2_ population. Functional validation was carried out through VIGS in the F_1_ (E3316) and overexpression performed in 19 141 and 19 147 for the two genes, respectively. We proposed that the *P* locus encodes an anthocyanidin synthase (ANS), and the *D* locus encodes an MYB1 transcription factor. Mutations of *SmANS* in 19 141 and *SmMYB1* in 19 147 may have resulted in the lack of anthocyanin biosynthesis in the fruit peel of these two parental lines. The cross between the two white-fruited parental lines would produce an F_1_ heterozygous at both loci, which suggests that the genes function complementarily, so that the F_1_ (E3316) fruits were purple red. In addition, due to the epistasis between these two candidate genes, the segregation ratio was 9:7 of plants with anthocyanin-pigmented fruits to these with white ones in the F_2_ population.

Therefore, this study analyzed the molecular mechanism of epistasis for regulation of eggplant fruit color, paving the way to identification and cloning of the other epistatic gene *Y*. Moreover, the two studied parental lines, as natural mutants, can be considered as ideal materials for studies on gene functions and breeding new varieties. From a plant production and breeding point of view, the developed molecular markers, *SmANS-Chr08SNP*, *SmMYB1-Chr10InDel1*, and *SmMYB1-Chr10InDel6* associated with the genes *P* and *D* in this study would aid in improving the fruit color trait of eggplant varieties.

## Materials and methods

### Plant materials

The two lines 19 147 and 19 141 were bred by the Institute of Vegetable Research, Guangdong Academy of Agricultural Sciences. The female parent 19 141 had white fruits with no anthocyanin in any plant part during the whole growth period. The fruits of the male parent 19 147 were also white, but the hypocotyl, leaf petioles, leaf main veins and flowers had purple or lavender colors. The line 19 147 was crossed to 19 141 to produce the F_2_ population. All of the 30 F_1_ (E3316) progeny exhibited purple red fruits, while a total of 1591 and 486 F_2_ progeny from two F_2_ populations showed extensive segregation of the fruit color phenotype, ranging from white to deep purple (Fig. 1). The ratio of plants with purple fruits to these with white fruits was 9:7 in the F_2_, suggesting that there were two pairs of epistatic genes (*D* and *P*) controlling the inheritance of eggplant fruit color [2].

### Phenotype evaluation of the F_2_ population

In 2015 and 2016, the parental lines, the F_1_ (E3316) and F_2_ populations were sown in a greenhouse, sequentially. The seedlings were planted in the field at the four-true leaf stage in the Tianhe District (113.35^°^N, 23.12°E), Guangdong, China. Although the degree of anthocyanin pigmentation can be affected by light, temperature, and other environmental factors, the presence or absence of anthocyanins was not affected by these environmental factors, and this phenotype was stable and consistent in different planting seasons. Thus, the fruit color phenotype of all the F_2_ progeny was recorded as anthocyanin pigmentation (scored as 1) or non-anthocyanin pigmentation (scored as 0) at the marketable fruit stage.

nthocyanins were extracted from peels of fruits of the parents and F_1_ (E3316) and then quantified using the Plant Anthocyanin Content Detection Kit (Solarbio, Beijing, China). The anthocyanin contents were calculated using the formula [45]: TA = [(A530nm-A620nm)-0.1(A650nm-A620nm)]/$\epsilon $ × (V/m) × M × 100, where TA is the total anthocyanin content (mg/100 g FW), $ \epsilon $ is the molar absorptivity, V is the total volume (ml), m is the sample weight (g), M is the molecular weight. Three biological replicates were used for each sample.

### Nucleic acid isolation and SLAF library construction

To prepare the SLAF library, genomic DNA was extracted from young leaves from the two parents and 200 F_2_ progeny plants (Table S2) using the cetyl trimethyl ammonium bromide (CTAB) method [46]. An improved SLAF-seq technology developed by Peking Biomarker Technology [24] was used for genotyping of all DNA samples. The assembled SME_ r2.5.1 genome sequence of eggplant [27] was used as a reference genome for a preliminary SLAF experiment to determine conditions and select restriction enzymes. Then *Rsa*I and *Hae*III were used to digest the genomic DNA. For RNA-seq, the total RNA of fruit peels was extracted from the parents and F_1_ (E3316) progeny from young and marketable fruits using TRIZOL kit (Thermo Fisher Scientific, USA). Then RNA was sequenced on the Illumina sequencing platform (IlluminaHiSeq™ 2000) using the paired-end technology.

### Map construction, QTL mapping and candidate gene analysis

The SLAF-seq data were processed as described by Sun et al. [24] and Wei et al. [47]. BWA [48] software was used to align the clean reads against the reference genome, and the SNPs detected by both GATK [49] and Samtools [50] were used as the final SNP dataset for further analysis. Only the marker type aa × bb was used to construct the genetic map for the F_2_ mapping population. To ensure the quality of LGs, SNPs were removed by three parameters including sequencing depth of two parental lines >4×, the coverage degree >70%, and the segregation distortion *p*-value<0.001. The high-quality SNPs were then used to construct the genetic map via HighMap [20] mapping software. SMOOTH [51] strategy and k-nearest neighbor algorithm were conducted to correct error and impute missing genotypes. Maximum likelihood [52] and Kosambi function [53] were used to add skewed markers and to estimate map distances, respectively.

For QTL analysis, the mapping data were obtained from the results of LG construction, and the fruit color records of 200 F_2_ progeny were used as phenotypic data. QTL mapping was conducted using QTL IciMapping V.3.3 software [54]. Inclusive composite interval mapping (ICIM) was applied for a one-dimensional scanning to detect additive and dominant QTL, while other parameters and LOD value (2.5) were set as defaults. Subsequently, a three-dimensional scanning was also conducted to detect digenic epistasis with QTL IciMapping V.3.3 software [54]. The ICIM-EPI (epistasis) mapping method was selected, and other parameters were set as defaults, including 25 cM as a step-in scanning.

According to the surrounding sequences of the QTL-flanking SNP markers, the corresponding QTL genomic regions were located on an eggplant reference genome GUIQIE-1 [21]. Then, the candidate genes associated with anthocyanin biosynthesis were searched according to the annotation information at the identified QTL regions on the reference genome.

### Quantitative real-time PCR (qRT-PCR) and phylogenetic analysis

Two DEGs related to anthocyanin biosynthesis were selected in the QTL regions and were verified by qRT-PCR. To analyze the transcript levels of the candidate genes in different tissues at the seedling and marketing stages, samples from roots, stems, leaves, petioles, leaf veins, flowers, peel and flesh of young fruits, as well as marketable ones, were collected with three biological replicates. Total RNA was extracted and cDNA synthesis and qRT-PCR with three technical replicates for each cDNA sample were performed using SYBR Premix Ex Taq Mix (TaKaRa, Japan) in the LightCycler 96 qRT-PCR Machine (Bio-Rad, USA). The primer sequences are provided in Table S8. The results were analyzed using 2^-ΔCt^ method, and the actin gene of eggplant was used as an internal control.

Protein sequences of *SmANS*, *SmMYB1* and the orthologs from *Solanum* and *Arabidopsis* were downloaded from NCBI (https://www.ncbi.nlm.nih.gov/protein). Then, two phylogenetic trees were constructed for *SmANS* orthologs and *SmMYB1* orthologs using the IQ tree [55] function in TBtools [56] with maximum likelihood statistical method and bootstrap analysis with 5000 replications.

### Investigation of genetic variation of *SmANS* and *SmMYB1* in the F_2_ population

A different set of 200 F_2_ plants was used to study the genetic variation of *SmANS* and *SmMYB1*. This included 50 plants with white flowers and white peels, 50 with purple flowers and white peels, and 100 with purple flowers and purple red peels. PARMS markers were developed to genotype the 200 F_2_ progeny at three variation loci of *SmANS* and *SmMYB1*. Primers for *SmANS-*Chr08SNP-F, *SmANS-*Chr08SNP-Rt, *SmANS-*Chr08SNP-Ra were used to genotype the *SNP* locus of *SmANS.* Primers for *SmMYB1-*Chr10InDel1-F, *SmMYB1-*Chr10InDel1-Ra, and *SmMYB1-*Chr10InDel1-Rc were used to genotype the G base InDel in the promoter of *SmMYB1*. Primers for *SmMYB1-*Chr10InDel6-Fa, *SmMYB1-*Chr10InDel6-Fg, and *SmMYB1-*Chr10InDel6-R were used to genotype the 6-bp InDel in R2 domain of *SmMYB1*. After PARMS-PCR reaction, the fluorescence signal of the PCR products was read using the TECAN infinite M1000 microplate reader, and analyzed and converted using the online software SNP decoder (http://www.snpway.com/snpdecoder/). The used primer sequences are provided in Table S8.

### *Agrobacterium* infiltration

Virus-induced gene silencing (VIGS) was performed using the tobacco rattle virus (TRV) according to the methods described previously [57]. Two fragments with 475 bp of *SmANS* (EGP18904) and 503 bp of *SmMYB1* (EGP21875) were amplified from 19 141 and 19 147 cDNAs, respectively, and then cloned into pTRV2 vectors to generate pTRV2-*SmANS* and pTRV2-*SmMYB* constructs. The recombinant plasmid was introduced into *Agrobacterium* strain GV3101 which was then cultured according to the method described by Wang and Fu [58]. After 10 h of shaking, the cells were centrifuged and re-suspended in the infiltration buffer. Suspensions of pTRV1 were mixed with pTRV2 and pTRV2-*SmANS* and pTRV2-*SmMYB1*, separately, at a ratio of 1:1 and maintained at 28°C for 3 h.

The *Agrobacterium* was infiltrated into eggplant fruits using a 1 mL-syringe, and pTRV2 was used as negative controls. Each inoculation was repeated three times for young fruits (7 days after pollination). After one week and when VIGS phenotype was observed, the fruits were collected to quantify the transcription levels of the target fragments in the fruit peels using qRT-PCR as described above. The used primer sequences are provided in Table S8.

### Vector construction and plant transformation

The full-length CDs of *SmANS* and *SmMYB1* were cloned into pEASY-T5 vector, transformed into T1 competent cells and sequenced using an Applied Biosystems 3500 genetic analyzer (Applied Biosystems, Shanghai, China) according to the manufacturer’s instructions (Sangon, Shanghai, China). The full-length (1242 bp) coding sequence of *SmANS* was amplified from 19 147 for *SmANS* and cloned into the Eco32I/ApaI sites of the pBWA(V)HS vector, driven by the CaMV 35S promoter. Meanwhile, to prepare the overexpression construct of *SmMYB1*, a 771 bp fragment containing the coding region was amplified from 19 141 (primers *SmMYB1*-F/R), and inserted into the Eco32I/ApaI sites of the pBWA(V)HS vector driven by the CaMV 35S promoter. Subsequently, these constructs as well as the empty one were transformed into *Agrobacterium rhizogenes* strain GV3101, which was previously used for VIGS. The lines 19 141 and 19 147 were used for generation of transgenic calli by Agrobacterium-mediated transformation. The transgenic calli were screened based on hygromycin resistance. The calli were induced in a growth chamber (16 h light at 28°C; 8 h dark at 25°C; light intensity of 45 μmol m^−2^ s^−1^). The calli induced after three weeks were used for observation of anthocyanin accumulation and analysis of the expression of *SmANS* and *SmMYB1*. The used primer sequences are provided in Table S8.

## Acknowledgments

The present study was financially supported by the National Natural Science Foundation of China (Grant No. 31501755), the Guangdong Provincial Natural Science Foundation (Grant No. 2021A1515012490), Major special projects of Guangxi science and technology program (AA22068088), the Department of agriculture and rural areas of Guangdong province of China, grant No. 2022KJ110 and 2022KJ106, and the Special fund for scientific innovation strategy-construction of high level Academy of Agriculture Science, grant number R2019PY-JX003, R2019PY-QF009, 202114TD, R2021YJ-YB3019. The authors would like to express their gratitude to EditSprings (https://www.editsprings.cn) for the expert linguistic services provided.

## Author contributions

S.B.J., L.Z.L., and S.G.W. conceived and designed the experiment. Y.Q. analyzed the data and wrote the manuscript. W.J., L.H.M and L.T conducted experiments, including sample collection, DNA and RNA extraction, qRT-PCR analysis, VIGS and overexpression. W.Y.K shared the GUIQIE1–1 reference genome and annotation files with us for this project. All authors read and approved the final manuscript.

## Data availability

All the raw sequencing data have been deposited at China National GeneBank (CNGB) database under Project accession number CNP0003597, Sample accession number CNS0623704-CNS0623905, and Experiment/Run accession number CNR0637917-CNR0638118.

## Conflict of interest

The authors declare no competing interests.

## Supplementary data

Supplementary data are available at *Horticulture Research* online.

## Supplementary Material

Web_Material_uhac268
