# Supplementary figures and images for: Mapping and validation of the epistatic D and P genes controlling anthocyanin biosynthesis in the peel of eggplant (Solanum melongena L.) fruit
Source: Hortic Res. 2022 Dec 2;10(2):uhac268. doi: 10.1093/hr/uhac268 (PMC9923212; doi:10.1093/hr/uhac268)

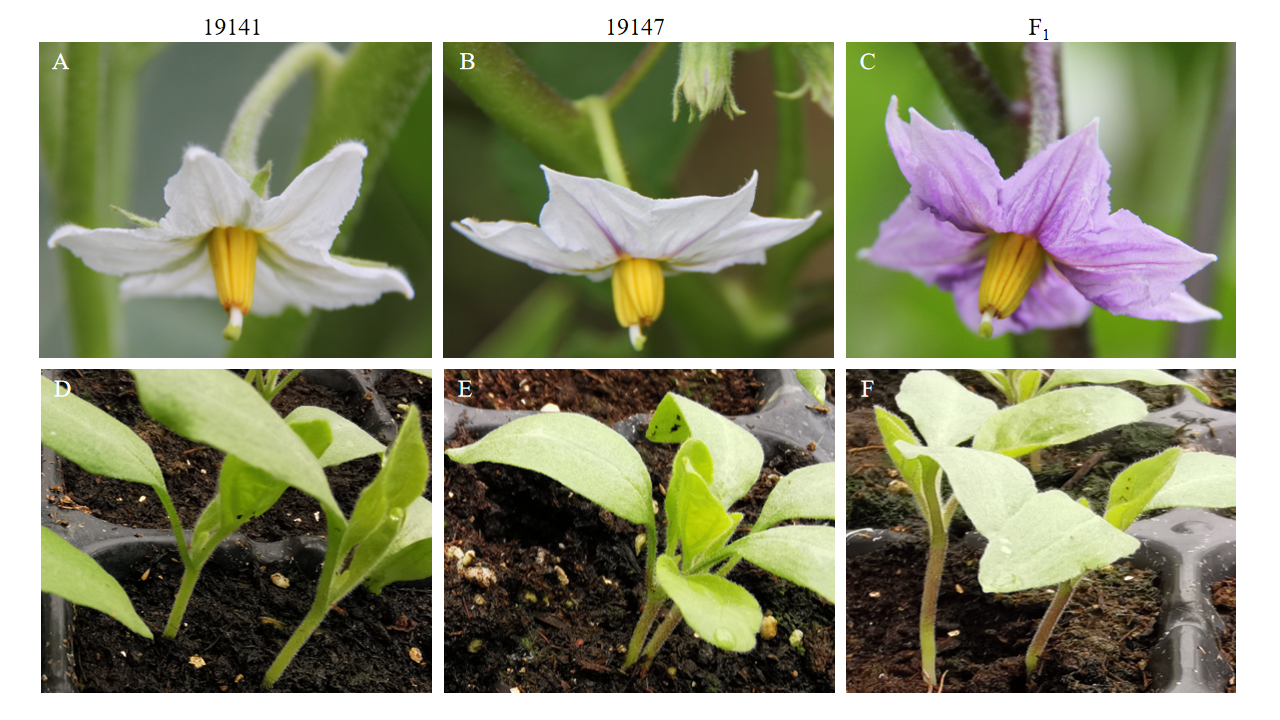

Supplement: Web_Material_uhac268 [file web_material_uhac268.zip › Fig. S1.tif]

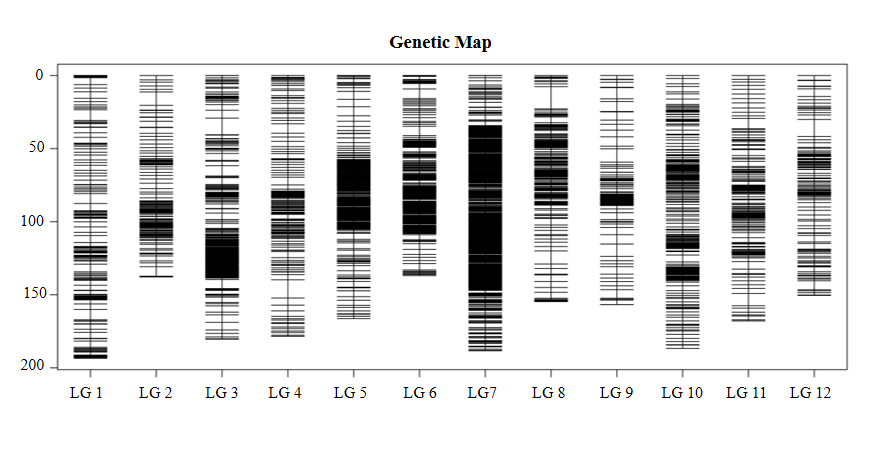

Supplement: Web_Material_uhac268 [file web_material_uhac268.zip › Fig. S2.tif]
